# Clinico-Epidemiological Analysis of Most Prevalent Parotid Gland Carcinomas in Poland over a 20-Year Period

**DOI:** 10.3390/ijerph191610247

**Published:** 2022-08-18

**Authors:** Michał Żurek, Kamil Jasak, Karolina Jaros, Piotr Daniel, Kazimierz Niemczyk, Anna Rzepakowska

**Affiliations:** 1 Department of Otorhinolaryngology Head and Neck Surgery, Medical University of Warsaw, 1a Banacha Str., 02-097 Warsaw, Poland; 2 Doctoral School, Medical University of Warsaw, 61 Żwirki i Wigury Str., 02-091 Warsaw, Poland; 3 Department of Analyses and Strategies, Ministry of Health, Miodowa 15 Str., 00-952 Warsaw, Poland; 4 Students Scientific Research Group at the Department of Otorhinolaryngology Head and Neck Surgery, Medical University of Warsaw, 1a Banacha Str., 02-097 Warsaw, Poland

**Keywords:** parotid gland cancer, mucoepidermoid carcinoma, adenoid cystic carcinoma, acinic cell carcinoma, adenocarcinoma, carcinoma in pleomorphic adenoma, squamous cell carcinoma, overall survival, clinico-epidemiological analysis

## Abstract

(1) Background: Malignant tumours of the salivary glands have different clinical and histopathological characteristics. They most commonly involve the parotid gland. Histopathologically, the most common are mucoepidermoid carcinoma (MEC), adenoid cystic carcinoma (AdCC), acinic cell carcinoma (AcCC), adenocarcinoma, carcinoma in pleomorphic adenoma (CPA), and squamous cell carcinoma (SCC). (2) Methods: We analysed 2318 patients with malignant parotid gland tumours reported to the National Cancer Registry (NCR) in Poland over 20 years (1999–2018). The demographic characteristics of patients, clinical factors, and overall survival (OS) were analysed. (3) Results: The average age was 61.33 ± 16.1 years. The majority were males (55%) and urban citizens (64%). High percentage of carcinomas was diagnosed in locoregional (33.7%) and systemic (10.4%) stadium. The most prevalent diagnoses were SCC (33.3%) and adenocarcinoma (19.6%). Surgical resection with adjuvant RT (42.1%) was the most common treatment. The OS analysis showed a median survival time of 5.6 years. The most favorable median OS was found in patients with AcCC (18.30 years), the worst for SCC (1.58 years). (4) Conclusion: AcCC has the best prognosis and SCC the worst. Tumour stadium, treatment, and demographic factors affect prognosis. Improvements in diagnosis and re-evaluation of treatment standards are necessary to enhance the outcome of patients with parotid gland cancers in Poland.

## 1. Introduction

Salivary gland tumours are heterogeneous lesions with complex clinicopathological characteristics. They constitute 3–10% of all head and neck cancers [1,2,3], in Europe the percentage amounts to 8.5% [4]. Tumours are mostly benign, only less than 20% of them are malignant [3,5]. The incidence rate of malignant neoplasms of the salivary glands depends on age and population, in most studies it amounts to 0.5–2/100,000 inhabitants [6,7,8], in Poland average incidence rate over last decade amounts to 1.78 [9]. The most common tumor location of salivary gland malignancies is the parotid gland, with a relative frequency of 58–69% [9,10,11]. Analyzing all lesions of parotid glands, malignant tumors comprise 15–32% of parotid tumors [8]. These data indicate the importance of parotid glands in the analysis of head and neck cancers, and therefore parotid gland carcinomas are the focus of this article.

The World Health Organization Classification of Head and Neck Tumours distinguishes 24 histopathological types of malignant epithelial tumours of salivary glands [12]. The most prevalent are mucoepidermoid carcinoma (MEC), adenoid cystic carcinoma (AdCC), acinic cell carcinoma (AcCC), and adenocarcinoma. Carcinoma ex pleomorphic adenoma (CPA) and squamous cell carcinoma (SCC) are less commonly diagnosed [8,13,14].

The risk factors of salivary gland cancers are still undefined. Some studies suggest that the history of head and neck cancer, radiotherapy, and some occupations were associated with an increased risk of salivary gland cancers [15,16]. A diet rich in fruit and vegetables and low in foods high in cholesterol may be effective in preventing salivary gland cancer [17]. The role of alcohol consumption and smoking in development of salivary gland cancer is still questionable [15,18,19].

Surgical resection represents the treatment of choice in malignant tumors of the salivary glands [20,21,22,23]. The method of surgery depends on the clinical characteristics of carcinoma. The role of elective neck dissection in every salivary gland carcinoma is still controversial, but it is recommended in patients with high-grade histology or T3/T4 tumors [21,23]. It is also recommended to supplement the resection with adjuvant radiotherapy (RT) in high grade or large tumors or in cases where there were incomplete or close resection margins [20,21,22,23,24]. Some authors recommend also adjuvant chemotherapy (CT) or radiochemotherapy (RCT) [22]. In the case of recurrent and inoperable tumors, it is recommended to consider palliative therapy including RT and/or CT [20].

The choice of appropriate treatment has a direct impact on patient prognosis, but it is not the only prognostic factor. Findings indicate clinical factors such as histopathological type and grade of lesion, advanced tumor stage, facial nerve involvement, vascular invasion, lymph node metastasis, and distant metastasis, negatively affect prognosis [14,25,26,27,28,29,30,31,32,33,34,35]. In addition, demographic factors such as gender or age are also factors that affect prognosis [14,26,31,34,36]. Assessing survival and risk of recurrence is extremely important for both clinicians and patients.

Epidemiological studies of the salivary glands pathologies are mainly focused on patients from one or few research centers. Our study covered patients from all over the country registered in The National Cancer Registry (NCR) over 20 years (1999–2018). This gives a comprehensive picture of specified types of carcinomas and applied treatment methods.

The aim of this study was to find out the relative frequency and clinical advancement of the selected, most often histological types of parotid gland cancers and to compare treatment modalities and determine overall survival and define risk factors for death in the Polish population between 1999 and 2018.

## 2. Materials and Methods

The study design was a nationwide and retrospective survey according to National Health Fund (NHF) and National Cancer Registry (NCR) databases [37,38]. The data concerns all patients who were diagnosed with parotid gland carcinoma in Poland between January 1999 and December 2018. The information includes medical data and demographical features, notably age of patients in moment of the diagnosis, area code, and sex of patients. Medical variables are histopathological diagnosis, disease stadium, treatment method, and survival time. 

The diagnoses are coded according to the International Classification of Diseases, 10th Revision (ICD-10) and the International Classification of Diseases for Oncology, 3rd Revision (ICD-O-3). During the study period each patient reported in databases with a confirmed primary diagnosis of parotid gland pathologies was retrospectively identified with ICD-10 codes C07 (with all extensions). The histopathological diagnoses were defined using ICD-O-3 codes:

- 8200/3 for adenoid cystic carcinoma (AdCC)
- 8430/3 for mucoepidermoid carcinoma (MEC)
- 8525/3, 8140/3 and 8147/3 for adenocarcinoma
- 8070/3, 8071/3, 8072/3 and 8073/3 for squamous cell carcinoma (SCC)
- 8550/3 for acinar cell carcinoma (AcCC)
- 6940/3 and 8914/3 for carcinoma in pleomorphic adenoma (CPA)

The treatment methods were identified in NCR database. All therapies are divided into the following groups: only surgery, only radiotherapy, surgery + radiotherapy, surgery + chemotherapy + radiotherapy, and other therapies. Other therapies included: chemoradiotherapy, immunotherapy, and other undefined therapies.

Cancer stadium is a simplified staging according to the clinical stage of disease in NCR reports. It is based on the TNM scale and consists of four levels: in situ, regional, loco-regional, and systemic stadium. Regional stage refers to T1–4 N0 M0, loco-regional to T1–4 N1–3 M0, and systemic stage to T1–4 N1–3 M1.

The information about deaths and population were obtained from Statistics Poland [39]. Patient deaths were reviewed until 31 December 2021.

A statistical analysis was performed for some demographic and clinical factors in the specified period. The following time intervals were created—from 1999 to 2003, from 2004 to 2008, from 2009 to 2013, and from 2014 to 2018. The same factors were also analysed in the specified parotid gland malignancy groups.

The next part of the study included survival analysis conducted in all patients diagnosed with selected types of parotid gland cancer during the period 1999–2018. The Kaplan–Meier curves were used to present the overall patient survival and log-rank tests were used to compare the groups depending on the selected variables (*p* values < 0.05 were considered statistically significant).

All statistical analyses were performed using R statistical software V. 3.6.2 (R Foundation for Statistical Computing, Vienna, Austria).

The study was consistent with the assumptions of the Declaration of Helsinki for human research. Demographic characteristics including age, gender, and place of residence were recorded anonymously. Conducting the research did not require the consent of the ethics committee due to the full anonymization of used data.

## 3. Results

Between 1999 and 2018, 2318 patients with specified parotid gland malignancies were registered. The number of patients significantly increased during the study period (*p* = 0.027). The average age of patients was 61.3 ± 16.1 years. The majority were males (55%) and urban citizens (64%). No statistically significant trend was observed for demographic data (Table 1).

Histopathologically, SCC constituted the largest group of malignancies (33.3%). This was followed by adenocarcinoma (19.6%), MEC (13.7%), and CPA (13.5%). AcCC accounted for the smallest histopathological group (8.6%). Analysis of the proportion of patients with SCC and CPA revealed a statistically significant increasing trend over the study period (*p* = 0.005, *p* = 0.026, respectively). The decreasing trend concerns patients with adenocarcinoma (*p* = 0.031). The mean age of patients at diagnosis date was the highest for SCC (67.6 ± 13.4) and the lowest for AcCC (54.1 ± 19.3). For AcCC, AdCC, and CPA, the majority of patients were female (64%, 59.6%, and 58.7% respectively). Regardless of the diagnosis, patients were predominantly urban residents (from 60.5% to 73.9%). The vast majority were regional stage tumours (56%), locoregional spread affected 33.7% of patients, and systemic spread 10.4%. The largest percentage of advanced stages was identified for SCC (47.4% patients for locoregional advancement and 13.1% for systemic) (Table 2).

**Figure 1:**
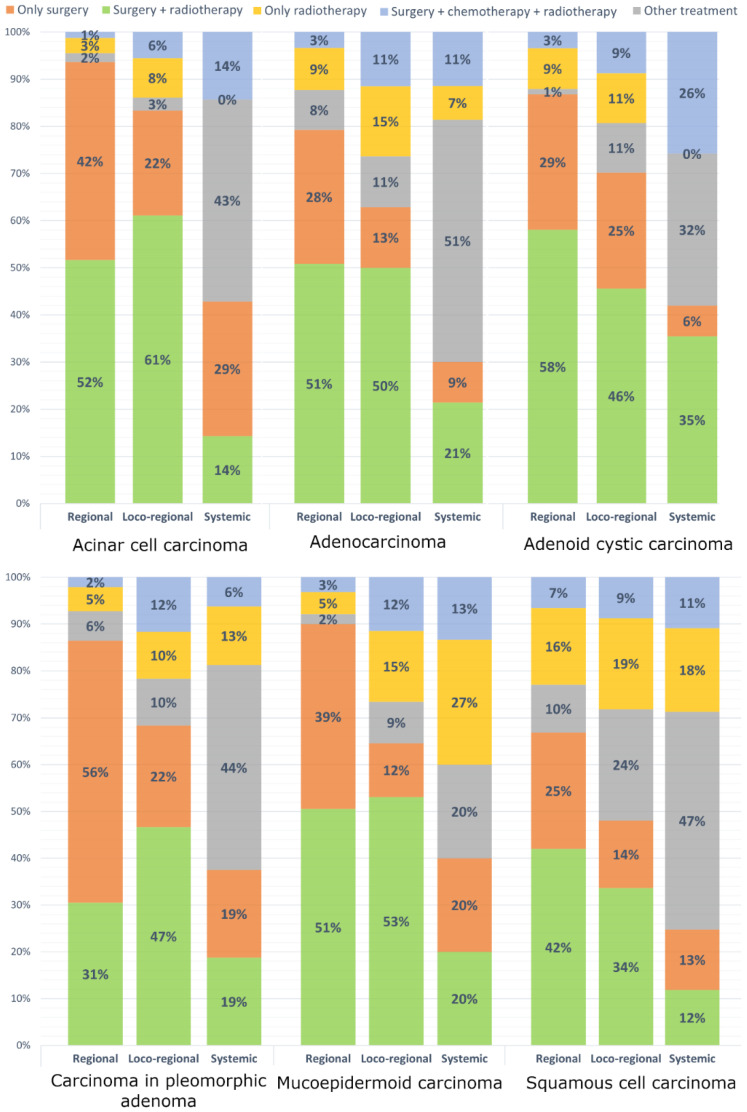
Frequency of treatment modalities stratified by stadium and histology of the cancer.

The most prevalent therapy for malignant parotid gland tumours was surgical resection with adjuvant RT (42.1%) and surgery alone (26.5%), and the least common was resection with RCT (6.6%). The type of therapy depended on the histopathological type and tumour stage (Figure 1). The fraction of patients treated with surgery alone and RT alone increased significantly (for both *p* < 0.001). However, the proportion of patients treated with surgery followed by RT or RCT decreased (*p* < 0.001, *p* = 0.022, respectively). Palliative therapies (defined as radiotherapy alone or other therapies) also showed an increasing trend (*p* < 0.001).

The OS analysis (Table 3) of selected parotid gland cancers showed a mean survival time of 5.6 years. The most favorable mean overall survival was found in patients with AcCC (18.3 years), CPA (17.4 years), and AdCC (14.9 years). More than 42% of patients survived 20 years or more. The shortest OS was for SCC with an average of 1.6 years; only 27.6% of patients survived 5 years. OS also decreases with tumour stadium (average 13.5 years OS for regional stage, 2.2 years for locoregional, and 1 for systemic). The therapy with the most favorable prognosis was surgery alone (average 15.1 years of OS) and surgery with radiotherapy (9.2 years of OS). The other therapies were associated with a worse prognosis.

**Figure 2:**
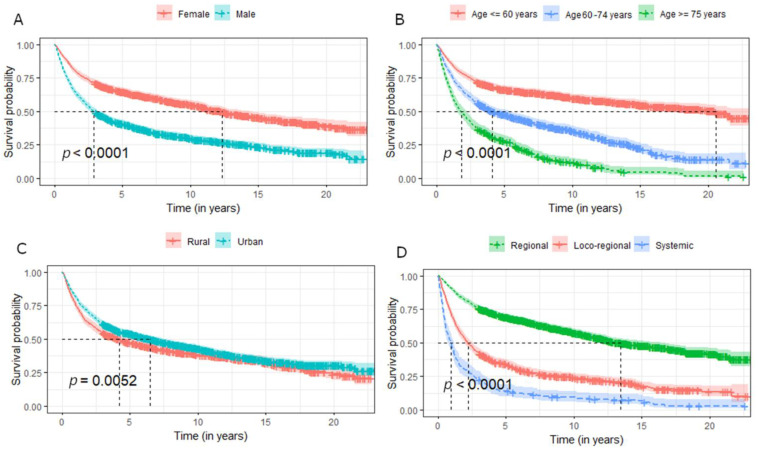
Survival of patients with parotid cancers stratified by demographic factors and stadium of the cancer: (**A**) sex, (**B**) group of age, (**C**) place of residence, (**D**) stadium.

**Figure 3:**
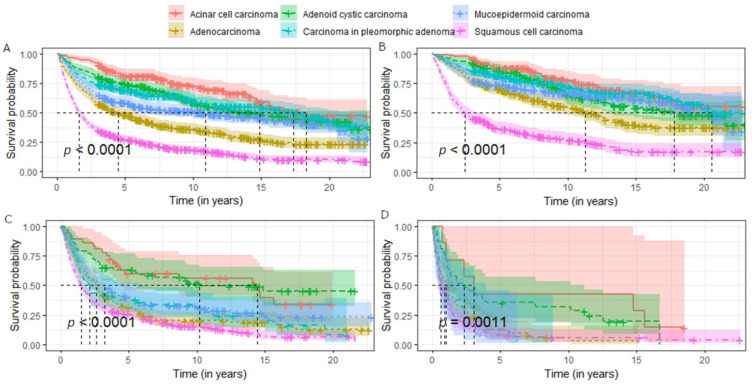
Survival of patients with specified parotid gland cancers stratified by stadium of advancement: (**A**) all cancers, (**B**)regional stadium, (**C**) locoregional stadium, (**D**) systemic stadium.

**Figure 4:**
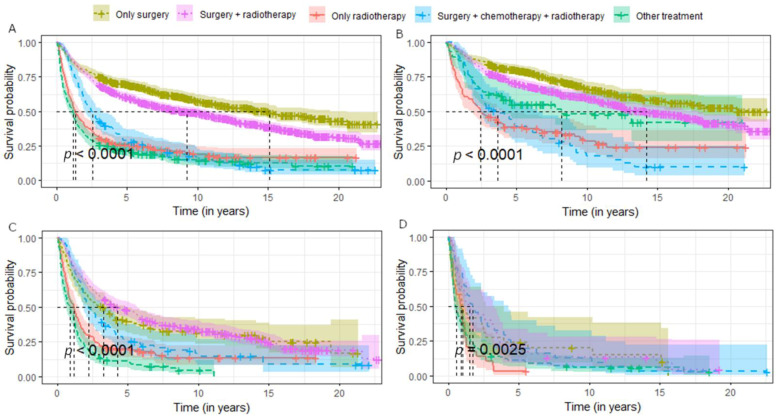
Survival of patients with parotid gland cancers stratified by treatment methods and stadium of the cancer: (**A**) all cancers, (**B**) regional stadium, (**C**) locoregional stadium, (**D**) systemic stadium.

The Kaplan–Meier curves presenting OS in patients with parotid cancers were stratified by demographic factors, stadium, histology, and treatment modality (Figure 2, Figure 3 and Figure 4).

## 4. Discussion

Salivary gland malignancies are a heterogeneous group of pathologies that vary significantly in terms of prognosis depending on the histopathological nature of the lesion. There have been several retrospective cohort studies so far [10,14,29,40,41,42,43], but none of them explored the issue in such a comprehensive way. To our knowledge, this is the only study that comprehensively assesses prognosis and risk factors in the six most common histopathological types of salivary gland malignancies. It is also one of the largest studies in terms of number of patients.

### 4.1. Epidemiological Characteristics of Patients

AcCC represented the youngest group among all tumours and the vast majority were women, which is consistent with literature data [31,42,44,45]. The demographic characteristics of patients with AdCC are consistent with other results in the literature, especially the gender ratio is consistent regardless of the centre or country studied (percentage of female patients between 59% and 67%) [46,47,48,49]. A systematic review including 13 articles and 263 patients with AdCC identified a mean patient age of 55.26 years and a female percentage of 56.5% [50]. CPA is a rare malignant tumour of the salivary glands. It represents 3–15% of all malignant salivary gland carcinomas [51], as in our study. The majority of studies report a higher incidence among men [33,51,52,53]. The range of patients mean age is from 57 to 62.1 years [33,51,52,53,54], which is consistent with our results. MEC is considered one of the most common salivary gland malignancies [11,41,55], however, in this study, MEC represents only 13.7% of all malignancies. Data from other studies confirm the average age of patients with MEC [41,55], but women appear to be more commonly affected than men [11,41,55,56,57], which is inconsistent with the above results. Adenocarcinoma is estimated also as one of the most common malignancies of the salivary glands and accounts for approximately 13–17% of all salivary carcinomas [58,59]. According to the literature, salivary gland adenocarcinoma more commonly affects men, with an average age range of 57.5–67 years [43,58,59,60]; our results confirm the literature data. Parotid gland SCC is thought to be more often metastatic cutaneous SCC rather than primary disease [40,61,62]. Generally it is thought to be a rare type among primary parotid pathology, however, it is considered highly malicious [61,63,64], but some epidemiological studies confirm the significant share of SCC among malignancies of parotid gland, especially when only metastases are analysed [65,66,67,68]. In the study of Mayer et al. [67], SCC accounted for 35.4% of all parotid malignancies, a very similar result to the above analysis. However, it should be emphasised that the vast majority of parotid SCCs are metastases from other sites, and they account for up to 86% of all salivary SCC [65,68]. The methodology of the study precludes the identification of primary and metastatic carcinomas, so the results reported concern both types of lesions (see “Limitations”). Epidemiological data indicate an approximately over 2-fold higher incidence among men [25,40,61,62,69]. The average age of patients is 64–73 years [40,61,69]. SCC is often diagnosed at the advanced stage with involvement of lymph nodes or with distant metastases [25,64]. The above results are consistent with those we presented.

In conclusion, the results are largely consistent with reports from other countries, demonstrating the lack of regional differences in demographic characteristics of patients with parotid gland malignancies. This allows us to conclude that geographical factors and related cultural differences do not influence the epidemiological depiction of parotid carcinomas.

### 4.2. Clinical Characteristics of Patients

AcCC is considered a slow-growing tumour and less aggressive than other malignancies [30,31,45,70], as confirmed by the above results. CPA is a rare and aggressive parotid carcinoma, and can develop de novo or based on pleomorphic adenoma. It is poorly understood and the clinical picture of the lesion cannot be briefly characterised; the exact factors for less or more aggressive course of CPA are not known [51,71,72]. Due to the high clinical variability at the time of diagnosis, the discrepancy in prognosis is so high. In the case of the present study, the majority of patients were in the early stages at the time of diagnosis, which should be considered as a coincidence or success of an effective diagnosis. For AdCC, the proportions in stages are similar to the average for all malignancies. Although these tumors have a slow growth rate, they are characterised by extensive local infiltration and a high risk of recurrence [47,49,73]. MEC is one of the most common major salivary malignancies [11,41,57]. Descriptions of MEC in the literature indicate a large variety of biological behavior and clinical course because of cellular heterogeneity. MEC can reoccur and metastasize to regional lymph nodes or distant sites [11,41,55,57], although in our study the vast majority of patients were diagnosed at the regional stadium of the disease. Adenocarcinoma (especially high-grade adenocarcinoma) presents aggressive features such as perineural invasion, positive margins, advanced T status, or lymph node involvement at the time of diagnosis [58,59]. Therefore, diagnosis is often made at an advanced stage, as confirmed by the results of our study. The higher frequency of diagnoses in advanced stage of cancer is also confirmed by Zhan et al. [43]. SCC of parotid glands are thought to be invasions from adjacent SCC of head and neck or distant metastases rather than de novo neoplasms; SCC arising de novo from the parotid gland comprise from 0.3 to 6.9% of primary salivary gland neoplasms [25,61,64]. Both primary parotid and metastatic SCC are aggressive with high malignant potential and the prognosis is relatively unfavorable [25,40,63,69]. Parotid SCC are usually diagnosed at an advanced stage with involvement of facial nerve and cervical metastases [25,62]. In our study, the data precludes to distinguish primary lesions from metastases, but still, the frequent diagnosis at an advanced stage and the poor prognosis are confirmed.

### 4.3. Treatment Modalities

The choice of therapy in malignancies of parotid glands depends mainly on the advancement of the disease and histopathological type of the lesion. According to NCCN Guidelines Version 2.2022 [24], surgery with complete resection of a tumour is the treatment of choice if there are no contraindications. In addition, postoperative RT should be considered in all cases of AdCC and for other malignancies when certain conditions are identified. RT is also recommended in most cases of recurrent lesions. New analyses highlight the role of RT in the treatment of salivary gland malignancies and indicate better OS in certain histopathological types of tumors [74].

Analysis of the treatment modalities in Poland shows a definite advantage of surgical treatment with RT in regional and locoregional stadium. Only for CPA at the initial stadium, the majority of patients (58%) were treated with surgery alone. NCCN guidelines do not indicate the need for specific treatment regimen in the case of CPA, however, some studies suggests that surgery followed by postoperative RT should be considered the standard of care [53,75]. The lack of an accurate clinical picture of the patients enrolled in the NCR database precludes to objectively assess the results presented; however, it is important to emphasise the current discrepancies in the results of efficacy of treatment modalities and to recommend the need for an individual approach and consideration of postoperative RT in each case.

A major deviation from current standards concerns patients with AdCC treated surgically without adjuvant RT. The current Polish recommendations are based on NCCN standards, but differ slightly from them. First of all, in the context of AdCC, surgical resection with postoperative RT is recommended when the lesion size exceeds 2 cm. This discrepancy may be one reason for such a high number of AdCC resections without RT. The majority of studies recommend surgery and postoperative RT for each primary AdCC, such treatment results in excellent outcomes with a low rate of late toxicity and preservation of a good quality of life [22,47,76].

The biggest deviation from the average treatment scheme concerns SCC. The overall analysis indicates the frequent use of palliative treatment methods. As stated in the previous paragraph, most parotid SCC lesions are invasions from adjacent cancers or distant metastases. In such cases, the use of palliative therapy is common. However, it is worth looking at the rather high percentage of RT alone. Studies on parotid SCC, however, show no benefit in the use of RT alone [40,62], even compared to no treatment at all [61]. It is therefore important to consider the precise indications for this type of treatment and the resulting benefits and disadvantages to the patient’s quality of life, especially with RT alone being so frequently chosen as a therapy.

Analysis of OS according to the performed therapy gives some unambiguous conclusions. Depending on the period analysed, the best prognostic therapy is surgery alone or surgery with adjuvant RT. Within 1 year from the diagnosis of the neoplasm, more patients survived when treated with surgery with RT comparing surgery alone, but in subsequent years this trend changes in favor of radical surgery. However, the results must be stratified by tumour stage in order to draw correct conclusions. In the regional stadium, average survival is better for surgical treatment alone, but in the loco-regional stage, surgery with adjuvant RT has a better prognosis for the first 10 years (Figure 4). The results are supported by other studies showing a lack of benefit of post-operative radiotherapy in early stages of salivary gland cancers [19,46].

### 4.4. Overal Survival

Other studies have reported higher 5-year OS than in our analysis (between 55 and 84.6%) but comparable 10-year OS (between 32 and 74.7%) [10,29,32,34,35]. The prognosis of OS for patients with parotid cancers depends on many factors. The major one is histopathologic type of the tumor.

AcCC is one with the most favourable prognosis. Positive results are confirmed in other studies, with 5- and 10-year survival rates ranging from 85 to 93% and from 79 to 88%, respectively [19,47,49,50,51].

Satisfactory prognosis concerns patients with AdCC, which is consistent with other studies with 5-year OS ranging from 67% to 92.5%, and 10-year OS from 25.6% to 65% [22,46,47,50,73,77,78].

The OS of CPA indicates a relatively good prognosis. The presented results are better than in other studies (range 25–68.5%) [33,51,53,54,71,72], but worse than in the study of Zbären et al. [79] who noted 5-year OS of 76%.

Results of OS for MEC are completely unsatisfactory compared to those reported in the literature. In the study by Boahene et al. [55], the 5- and 10-year OS were 96.6% and 97.4%, respectively, and in the study by Chan et al. [11] 93.6% and 67.4%, respectively. In the US, the 5-year OS was determined as 75.2% [41].

Poor prognosis concerns patients with adenocarcinoma, which was confirmed by other studies; 5-year OS was estimated at 43–62.2% [43,58,59,60,72]; and 10-year OS was only 20.7% [58].

SCC is one of the cancers with the worst prognosis. The 5-year survival is estimated at 25–31% [63,64], which is consistent with the results of the above analysis.

In the case of AcCC and MEC, the prognosis is generally worse than in other studies. The reason for differences in MEC may be due to the rather high proportion of patients with advanced stage of the disease compared to proportions from the cited studies. In the case of AcCC, most of the reported cancers were in regional stadium, so there is no clear justification for the difference.

Presented outcomes analysis shows that there is a potential to improve the prognosis of patients with parotid gland cancers in Poland both through more effective and earlier initiated diagnostics and application of more comprehensive treatment modalities.

### 4.5. Limitations

This study analyses cases registered in the NCR database. This is a nationwide database, but due to the dispersed nature of the reporting, we have no assurance of the reliability and completeness of the data and the results may be subject to error, which is beyond the control of the authors. The NCR database contains specific clinical data with varying degrees of detail. In the above analysis, we could differentiate regional, local, or distant disease. More detailed data on the TNM classification or clinical stage were highly incomplete, which precluded an accurate statistical analysis. The lack of clinical data on individual patients is a significant limitation of the study, the reason is the specific data encoding profile in the NCR and NHF databases and the authors of the project have no influence on the issue.

The evaluation of parotid SCC in the study group requires separate comment. The significant proportion of this type of cancer is probably due to the presence of metastatic lesions in the salivary glands. The data provided did not include information on other types of cancer, so we cannot identify which patients have SCC as a primary cancer or as a metastasis. This is a significant limitation in the clinical interpretation of the results. However, it should be noted that primary parotid SCC is a diagnosis by exclusion, and with this histopathological diagnosis, it should be assumed that the lesion is a metastasis and the primary cancer should be sought.

## 5. Conclusions

Over the 20 years there were no observed changes in the age, sex, and inhabitant of patients with the analysed malignant parotid gland neoplasms. The results of demographic analyses are largely consistent with reports from other countries, demonstrating the lack of regional differences in demographic characteristics of patients with parotid gland malignancies. Treatment modalities for malignancies of parotid glands do not follow NCCN standards in every case; the high proportion of patients treated with RT alone remains controversial and requires detailed reevaluation regarding presented poor survival outcomes for this modality. SCC constituted the largest group of malignancies (33.3%), followed by adenocarcinoma (19.6%) and MEC (13.7%). SCC include primary or metastatic tumours, thereby results in this group concern a heterogeneous group of patients. The best performing malignancy among those analysed is AcCC, and the worst is SCC. Tumour stadium seems to have the crucial role when assessing prognosis.

Presented analysis shows potential directions to improve the outcomes in patients with parotid gland cancers in Poland.

## Figures and Tables

**Table 1:** Characteristics of patients diagnosed with parotid cancers in NCR in 1999–2018.

Years	1999–2003	2004–2008	2009–2013	2014–2018	All	*p*-Value
Total number of selected parotid gland cancers	460	548	644	666	2318	0.027
Demographic characteristics						
Mean age ± SD	57.9 ± 15.2	60.4 ± 16.5	63.1 ± 15.7	62.8 ± 16.4	61.3 ± 16.1	0.075
Women (%)	209 (45.4%)	248 (45.3%)	274 (42.6%)	313 (47%)	1044 (45%)	0.442
Men (%)	251 (54.6%)	300 (54.7%)	370 (57.4%)	353 (53%)	1274 (55%)	
Urban citizens (%)	311 (67.6%)	340 (62%)	404 (62.7%)	430 (64.6%)	1485 (64%)	0.256
Rural citizens (%)	149 (32.4%)	208 (38%)	240 (37.3%)	236 (35.4%)	833 (36%)	
Histopathological diagnosis						
Adenoid cystic carcinoma	63 (13.7%)	62 (11.3%)	75 (11.7%)	62 (9.3%)	262 (11.3%)	0.148
Mucoepidermoid carcinoma	57 (12.4%)	87 (15.9%)	93 (14.4%)	81 (12.2%)	318 (13.7%)	0.214
Adenocarcinoma	104 (22.6%)	118 (21.5%)	104 (16.2%)	128 (19.2%)	454 (19.6%)	0.031
Squamous cell carcinoma	131 (28.5%)	168 (30.7%)	244 (37.9%)	229 (34.4%)	772 (33.3%)	0.005
Acinar cell carcinoma	30 (6.5%)	45 (8.2%)	59 (9.2%)	66 (9.9%)	200 (8.6%)	0.228
Carcinoma in pleomorphic adenoma	75 (16.3%)	68 (12.4%)	69 (10.7%)	100 (15%)	312 (13.5%)	0.026
Clinical stage						
Regional	264 (57.4%)	308 (56.2%)	342 (53.1%)	384 (57.7%)	1298 (56%)	0.347
Locoregional	156 (33.9%)	181 (33%)	231 (35.9%)	212 (31.8%)	780 (33.6%)	0.472
Systemic	40 (8.7%)	59 (10.8%)	71 (11%)	70 (10.5%)	240 (10.4%)	0.616
Therapy						
Only surgery	107 (23.3%)	127 (23.2%)	162 (25.2%)	219 (32.9%)	615 (26.5%)	<0.001
Only radiotherapy	32 (7%)	48 (8.8%)	91 (14.1%)	95 (14.3%)	266 (11.5%)	<0.001
Surgery + radiotherapy	243 (52.8%)	274 (50%)	259 (40.2%)	200 (30%)	976 (42.1%)	<0.001
Surgery + chemotherapy + radiotherapy	37 (8%)	38 (6.9%)	51 (7.9%)	28 (4.2%)	154 (6.6%)	0.022
Others	41 (8.9%)	61 (11.1%)	81 (12.6%)	124 (18.6%)	307 (13.2%)	<0.001

**Table 2:** Characteristic of different histopathological types of parotid cancers.

Histopathology	Adenoid Cystic Carcinoma	Mucoepidermoid Carcinoma	Adenocarcinoma	Squamous Cell Carcinoma	Acinar Cell Carcinoma	Carcinoma in Pleomorphic Adenoma	All
All patients	262	318	454	772	200	312	2318
Demographic characteristics							
Mean age ± SD	56.34 ± 15.9	56.4 ± 18.6	62.9 ± 13.3	67.6 ± 13.4	54.1 ± 19.3	57.4 ± 15.3	61.3 ± 16.1
Women	155 (59.2%)	146 (45.9%)	184 (40.5%)	248 (32.1%)	128 (64%)	183 (58.7%)	1044 (45%)
Men	107 (40.8%)	172 (54.1%)	270 (59.5%)	524 (67.9%)	72 (36%)	129 (41.3%)	1274 (55%)
Urban citizens	192 (73.9%)	205 (64.5%)	295 (65%)	470 (60.9%)	121 (60.5%)	202 (64.7%)	1485 (64.1%)
Rural citizens	70 (26.1%)	113 (35.5%)	159 (35%)	302 (39.1%)	79 (39.5%)	110 (35.3%)	833 (35.9%)
Clinical stage							
Regional	174 (66.4%)	190 (59.8%)	236 (52%)	305 (39.5%)	157 (78.5%)	236 (75.6%)	1298 (56%)
Locoregional	57 (21.8%)	113 (35.5%)	148 (32.6%)	366 (47.4%)	36 (18%)	60 (19.2%)	780 (33.8%)
Systemic	31 (11.8%)	15 (4.7%)	70 (15.4%)	101 (13.1%)	7 (3.5%)	13 (4.2%)	237 (10.2%)
Therapy							
Only surgery	66 (25.4%)	91 (28.6%)	92 (20.3%)	142 (18.4%)	76 (38%)	148 (47.4%)	615 (26.5%)
Only RT	21 (8.1%)	30 (9.4%)	48 (10.6%)	139 (18%)	8 (4%)	20 (6.4%)	266 (11.5%)
Surgery + RT	138 (53.1%)	159 (50%)	209 (46%)	263 (34.1%)	104 (52%)	103 (33%)	976 (42.1%)
Surgery + RCT	19 (7.3%)	21 (6.6%)	33 (7.3%)	63 (8.2%)	5 (2.5%)	13 (4.2%)	154 (6.6%)
Others	18 (3.9%)	17 (3.1%)	72 (11.2%)	165 (24.8%)	7 (0.3%)	28 (1.2%)	307 (13.2%)

**Table 3:** OS of patients with parotid cancers stratified by histology, stadium, and therapy.

OS	Median (Years)	1-Year (%)	5-Years (%)	10-Years (%)	20-Years (%)
All analysed parotid gland cancers	5.6	80%	51.7%	41.1%	28.1%
Histopathological diagnosis					
Acinar cell carcinoma	18.3	97%	81.1%	72.6%	47.3%
Adenocarcinoma	4.5	81.3%	48.7%	35.9%	23.6%
Adenoid cystic carcinoma	14.9	91.6%	75%	58.4%	42.7%
Carcinoma in pleomorphic adenoma	17.4	88.1%	69.6%	60.7%	42.2%
Mucoepidermoid carcinoma	10.9	85.8%	58.9%	51.2%	40.2%
Squamous cell carcinoma	1.6	65.2%	27.6%	18.5%	10.3%
Clinical stage					
Regional	13.5	90.8%	69.3%	57.5%	41.8%
Locoregional	2.21	71.4%	34%	24.1%	13.5%
Systemic	1	49.6%	14%	9.4%	2.3%
Therapy					
Only surgery	15.1	86.8%	69.6%	57.5%	43.5%
Only radiotherapy	1.3	59%	26%	18.7%	16.9%
Surgery + radiotherapy	9.2	89.2%	60.5%	49.2%	31.7%
Surgery + chemotherapy + radiotherapy	2.5	83.8%	28.4%	15.2%	7.8%
Other treatment	1.2	53.1%	21.8%	14.8%	10.5%

## Data Availability

The National Cancer Registry data are available at http://onkologia.org.pl/ (accessed on 1 April 2022) and the Statistics Poland data are available at http://www.stat.gov.pl (accessed on 1 April 2022).

## Abbreviations

AcCC	acinic cell carcinoma
AdCC	adenoid cystic carcinoma
CPA	carcinoma in pleomorphic adenoma
CT	chemotherapy
MEC	mucoepidermoid carcinoma
NCR	National Cancer Registry
NHF	National Health Fund
OS	overall survival
RCT	radiochemotherapy
RT	radiotherapy
SCC	squamous cell carcinoma
